# Potentially Inappropriate Medication Use in Older Adults in Primary Care According to Four Explicit Criteria: Associations with Patient Factors

**DOI:** 10.3390/medicina62091813

**Published:** 2026-09-20

**Authors:** Mihaela Paraschiv, Alexandru Vasincu, Răzvan-Nicolae Rusu, Oana Irina Gavril, Bogdan Andrei Trandabăț, Andrei Ciobîcă, Veronica Bild

**Affiliations:** 1 Discipline of Pharmacodynamics and Clinical Pharmacy, Department of Pharmaceutical Sciences II, Faculty of Pharmacy, “Grigore T. Popa” University of Medicine and Pharmacy, 700115 Iasi, Romania; paraschiv_mihaela@d.umfiasi.ro (M.P.); alexandru.vasincu@umfiasi.ro (A.V.); veronica.bild@umfiasi.ro (V.B.); 2 Department of Medical Specialties I, Faculty of Medicine, “Grigore T. Popa” University of Medicine and Pharmacy, 700115 Iasi, Romania; oana-irina.gavril@umfiasi.ro; 3 Department of Surgery II, Faculty of Medicine, “Grigore T. Popa” University of Medicine and Pharmacy, 700115 Iasi, Romania; trandabat.bogdan-andrei@d.umfiasi.ro; 4 Romanian Association of Algesiology, 700117 Iasi, Romania; andrei.ciobica@yahoo.com; 5 “Olga Necrasov” Center of Anthropological and Biomedical Research, Romanian Academy, Iasi Branch, 700506 Iasi, Romania

**Keywords:** potentially inappropriate medication list, inappropriate prescribing, older adults, primary health care, STOPP criteria, Beers Criteria, obesity, polypharmacy, medication burden

## Abstract

*Background and Objectives*: Potentially inappropriate medication (PIM) use is associated with adverse clinical outcomes in older adults. Several explicit screening tools are available for identifying PIMs, but Romanian data regarding the application of updated criteria remain limited. This study aimed to assess PIM identification according to the STOPP v3, Beers, EU(7)-PIM criteria and PRISCUS v2, to compare PIM counts and PIM presence across criteria, and to explore associations between PIM counts and selected patient characteristics. *Materials and Methods*: A retrospective observational study with a cross-sectional design was conducted in a single family medicine practice in Iasi, Romania, including 100 patients aged ≥ 65 years with at least one chronic condition and receiving at least two medications. Medications documented between January and June 2024 were assessed using the four explicit PIM criteria. One-way ANOVA with Bonferroni correction, repeated-measures ANOVA, agreement analyses, and Poisson regression models were used to examine differences between criteria and associations with patient characteristics, multimorbidity, and medication burden. *Results*: Mean PIM counts ranged from 0.75 for PRISCUS to 1.31 for STOPP. At least one PIM was identified in 73% of participants according to STOPP, 62% according to Beers, 60% according to EU(7)-PIM, and 53% according to PRISCUS. STOPP identified significantly more PIMs than each of the other three criteria (*p* < 0.001), while pairwise kappa coefficients ranged from 0.506 to 0.777. Among the 16 one-way ANOVA comparisons, only the association between obesity and EU(7)-PIM counts remained significant after Bonferroni correction (*p* < 0.001). In adjusted Poisson models, medication burden was associated with PIM counts according to STOPP (*p* < 0.001), Beers (*p* = 0.016), and EU(7)-PIM (*p* = 0.018). Obesity remained associated with EU(7)-PIM counts (*p* = 0.001), multimorbidity with STOPP PIM counts (*p* = 0.049), and sex with PRISCUS PIM counts (*p* = 0.030). Age, type 2 diabetes mellitus, and dyslipidemia were not independently associated with PIM counts. *Conclusions*: PIM identification varied according to the explicit criteria applied, with STOPP identifying the highest mean PIM count. Medication burden was the factor most consistently associated with PIM counts in adjusted analyses, while other patient characteristics showed criterion-specific associations. Given the small sample, single-practice setting, and non-probability sampling approach, these findings should be considered exploratory and require confirmation in larger, multicenter primary care studies.

## 1. Introduction

Irrational use of medicines represents a major global health challenge, with significant clinical and economic consequences. The World Health Organization estimates that over 50% of medicines are prescribed, dispensed, or sold inappropriately and that a similar proportion of patients do not use them as directed, leading to unnecessary healthcare resource utilization and increased public health risks [1].

Potentially inappropriate prescribing (PIP) refers to prescribing practices that may expose older adults to an increased risk of medication-related adverse outcomes [2]. PIP encompasses potentially inappropriate medications (PIMs), for which the risks may outweigh the benefits, particularly when safer therapeutic alternatives are available, and potential prescribing omissions (PPOs), referring to the omission of clinically indicated medications [3].

Older adults with multimorbidity are frequently exposed to polypharmacy, increasing the risk of PIP due to competing disease-specific guidelines, drug–drug interactions, and age-related pharmacokinetic and pharmacodynamic changes [4,5].

PIM use in older adults has been associated with several adverse clinical outcomes. Evidence from systematic reviews and meta-analyses indicates that PIM exposure is linked to an increased risk of adverse drug reactions and hospitalization, as well as higher rates of emergency department visits and healthcare utilization [6,7,8]. In addition, longitudinal and cohort studies have described associations between PIM use and higher risk of falls, functional decline, and mortality, highlighting the substantial clinical burden of inappropriate prescribing among older adults [9,10].

Various strategies have been developed to identify and reduce PIM use in older adults, including implicit methods based on clinical judgment and explicit criteria-based tools [11]. Widely used explicit tools include the STOPP/START criteria [12], the Beers Criteria [13], the EU(7)-PIM list [14], and PRISCUS [15]. STOPP/START criteria are among the most validated tools in European populations, allowing for the assessment of both PIMs and prescribing omissions [11], while the Beers Criteria remain the primary reference in the United States [11,13]. Evidence suggests that combining multiple screening tools improves the detection of potentially inappropriate prescribing in older adults [16].

Demographic aging further intensifies this issue. Globally, the population aged 65 years and older is projected to double from 703 million in 2019 to 1.5 billion by 2050, contributing to higher rates of multimorbidity, polypharmacy, and PIM exposure [17,18,19].

Romania follows similar demographic trends, with the proportion of adults aged over 65 years expected to reach approximately 31% by 2050 [20]. As multimorbidity remains highly prevalent among older adults, the need for pharmacological treatment increases, consequently raising the risk of polypharmacy and potentially inappropriate prescribing [20].

The prevalence of PIM use varies considerably across studies and countries, ranging from 8.6% to over 80%, with most studies reporting values around 30% [21]. Differences in study populations, healthcare settings, and explicit screening criteria contribute substantially to this variability. In Europe, high PIM prevalence rates have been reported in primary care settings, including 21.4% in Ireland [22], 40.4% in Spain [23], and 76.7% in Switzerland [24].

Romanian data show a similar pattern, although estimates vary by care setting. Reported PIM prevalence ranges from 18.58% to 25.80% in ambulatory populations [25,26], while a Romanian nursing-home study found that 69.23% of residents had at least one misuse-related PIP [27]. These findings suggest that a considerable proportion of older adults, particularly those exposed to polypharmacy, may benefit from systematic medication review based on explicit screening tools.

Given the limited Romanian data on the application of updated explicit PIM criteria and the clinical burden associated with PIM use, further research is needed. This study aimed to assess PIM identification according to the STOPP v3, Beers, EU(7)-PIM criteria and PRISCUS v2, compare the mean number of PIMs identified per patient by each tool, and examine the associations between PIM counts and selected demographic and clinical characteristics, including age, sex, obesity, type 2 diabetes mellitus, and dyslipidemia.

## 2. Materials and Methods

### 2.1. Study Design and Setting

A retrospective observational study with a cross-sectional design was conducted in a family medicine practice in Iasi, a city located in north-eastern Romania.

### 2.2. Study Population and Participant Selection

The study included patients aged 65 years or older with at least one documented chronic condition. To be eligible, patients were required to have attended at least one consultation with the family physician between January and June 2024, to have at least one documented visit in 2023, and to be receiving treatment with at least two medications. Patients with terminal-stage disease were excluded.

Before data collection, predefined eligibility criteria were established by the investigator and communicated to the family physician. The family physician identified medical records meeting the predefined eligibility criteria and provided eligible records sequentially until the target sample of 100 patients was reached. This constituted a non-probability sampling approach. The exact number of patients meeting all eligibility criteria was not prospectively recorded.

The target sample of 100 patients was determined pragmatically based on the feasibility of manual medical-record review and the availability of eligible records; no formal a priori sample-size calculation was performed. All 100 included patients were retained in the final analytical sample, with no subsequent participant-level exclusions.

Patients receiving only one medication were excluded because the database was designed to evaluate prescribing practices and medication-related safety issues in older adults receiving more than one pharmacological treatment, including PIM use and subsequent analyses of potential drug–drug interactions.

### 2.3. Data Collection

Data were collected from patients’ medical records, including consultation records and attached specialist referral letters, and were manually extracted from paper-based documents by a clinical pharmacist between 15 October 2024 and 17 June 2025. The analysis included medications prescribed and documented in the patients’ medical records during the study period (January–June 2024).

### 2.4. Study Variables and PIM Assessment

Collected data included sociodemographic variables (age and sex), clinical diagnoses, and detailed information on prescribed medications, including international nonproprietary name (INN), dose, route of administration, regimen, and duration of treatment. Topical medications were not included in the analysis. The number of distinct chronic diagnoses documented for each patient was calculated and used as an indicator of multimorbidity. Medication burden was represented by the number of distinct medications per patient during the study period.

Potentially inappropriate medications (PIMs) were identified using four explicit screening tools: Screening Tool of Older Persons’ Prescriptions (STOPP) version 3 [12], PRISCUS version 2 [15], the Beers Criteria [13], and the EU(7)-PIM list [14]. The number of PIMs identified for each patient was calculated according to each set of criteria. PIM identification was performed by a single clinical pharmacist based on the documented clinical diagnoses and medication-related information available in the patients’ medical records.

Selected chronic conditions, including obesity, type 2 diabetes mellitus, and dyslipidemia, were analyzed as potential factors associated with PIM counts. These conditions were identified based on diagnoses documented in the patients’ family medicine medical records; obesity was not reclassified using a study-defined BMI threshold.

### 2.5. Statistical Analysis

Descriptive statistics were used to summarize participant characteristics, PIM counts, and PIM presence. Continuous variables were expressed as means, standard deviations (SDs), and ranges, while categorical variables were presented as frequencies and percentages, as appropriate.

One-way analysis of variance (ANOVA) was used to compare mean PIM counts between groups defined by dyslipidemia, obesity, type 2 diabetes mellitus, and sex for each of the four explicit PIM criteria. A Bonferroni correction was applied to account for multiple comparisons across the 16 ANOVA analyses.

As all four PIM criteria were applied to the same participants, repeated-measures ANOVA was used to compare PIM counts across criteria. Sphericity was assessed using Mauchly’s test, with the Greenhouse–Geisser correction applied when appropriate. Pairwise comparisons were adjusted using the Bonferroni method. The proportion of participants with at least one PIM and the overlap between criteria were also examined, while pairwise agreement was assessed using Cohen’s kappa.

Given the count nature of the PIM outcomes, Poisson regression models were considered the primary regression analyses. Separate models were used to examine associations between selected patient characteristics and PIM counts and the relationship between medication burden and PIM counts. Adjusted Poisson regression models included obesity, type 2 diabetes mellitus, dyslipidemia, sex, age, multimorbidity, and medication burden. Model fit was assessed using deviance and Pearson chi-square statistics. Linear regression analyses were retained as supplementary sensitivity analyses: simple linear regression was used to examine the association between age and PIM counts, while multivariable linear regression models examined associations between obesity, type 2 diabetes mellitus, dyslipidemia, sex, and PIM counts. A bootstrap sensitivity analysis based on 1000 resamples was also performed for the mean EU(7)-PIM count among participants with obesity.

Statistical analyses were performed using Statistical Package for the Social Sciences (SPSS), version 28. Statistical significance was set at *p* < 0.05, with Bonferroni adjustment applied to the 16 one-way ANOVA comparisons. Also, 95% confidence intervals were reported where appropriate.

## 3. Results

### 3.1. Descriptive Characteristics

Descriptive statistics were calculated for participant characteristics and the number of potentially inappropriate medications (PIMs) identified by each tool. The sample consisted of 100 older adults with a mean age of 72.93 years (SD = 5.46, range = 65–91). The sample comprised 44 males and 56 females. The mean (SD) number of distinct chronic diagnoses per patient was 5.04 (SD = 2.93; range, 1–14) and the mean number of distinct medications per patient was 8.86 (SD = 3.85; range, 2–21). Obesity was documented in 11% of participants, type 2 diabetes mellitus in 25%, and dyslipidemia in 42%. The number of PIMs identified by the STOPP criteria ranged from 0 to 4, with a mean of 1.31 (SD = 1.05). Using the Beers Criteria, the number of PIMs ranged from 0 to 4, with a mean of 0.91 (SD = 0.91). The EU(7)-PIM tool identified between 0 and 4 PIMs per participant, with a mean of 0.90 (SD = 0.93), whereas the PRISCUS list identified between 0 and 4 PIMs per participant, with a mean of 0.75 (SD = 0.89).

### 3.2. Associations Between Dyslipidemia and PIM Counts

#### 3.2.1. STOPP Criteria

A one-way ANOVA was conducted to examine differences in STOPP PIM counts between participants with and without dyslipidemia. The analysis showed no statistically significant effect of dyslipidemia on STOPP PIMs, F(1, 98) = 3.06, *p* = 0.083. Although the dyslipidemia group had a higher mean number of STOPP PIMs (M = 1.52, SD = 1.15) compared to those without dyslipidemia (M = 1.16, SD = 0.95), this difference did not reach statistical significance.

#### 3.2.2. Beers Criteria

An ANOVA assessing Beers’ PIM counts indicated no significant difference between groups, F(1, 98) = 1.13, *p* = 0.290. Participants with dyslipidemia (M = 1.02, SD = 1.00) did not differ meaningfully from those without dyslipidemia (M = 0.83, SD = 0.84).

#### 3.2.3. EU(7)-PIM Criteria

**Figure 1:**
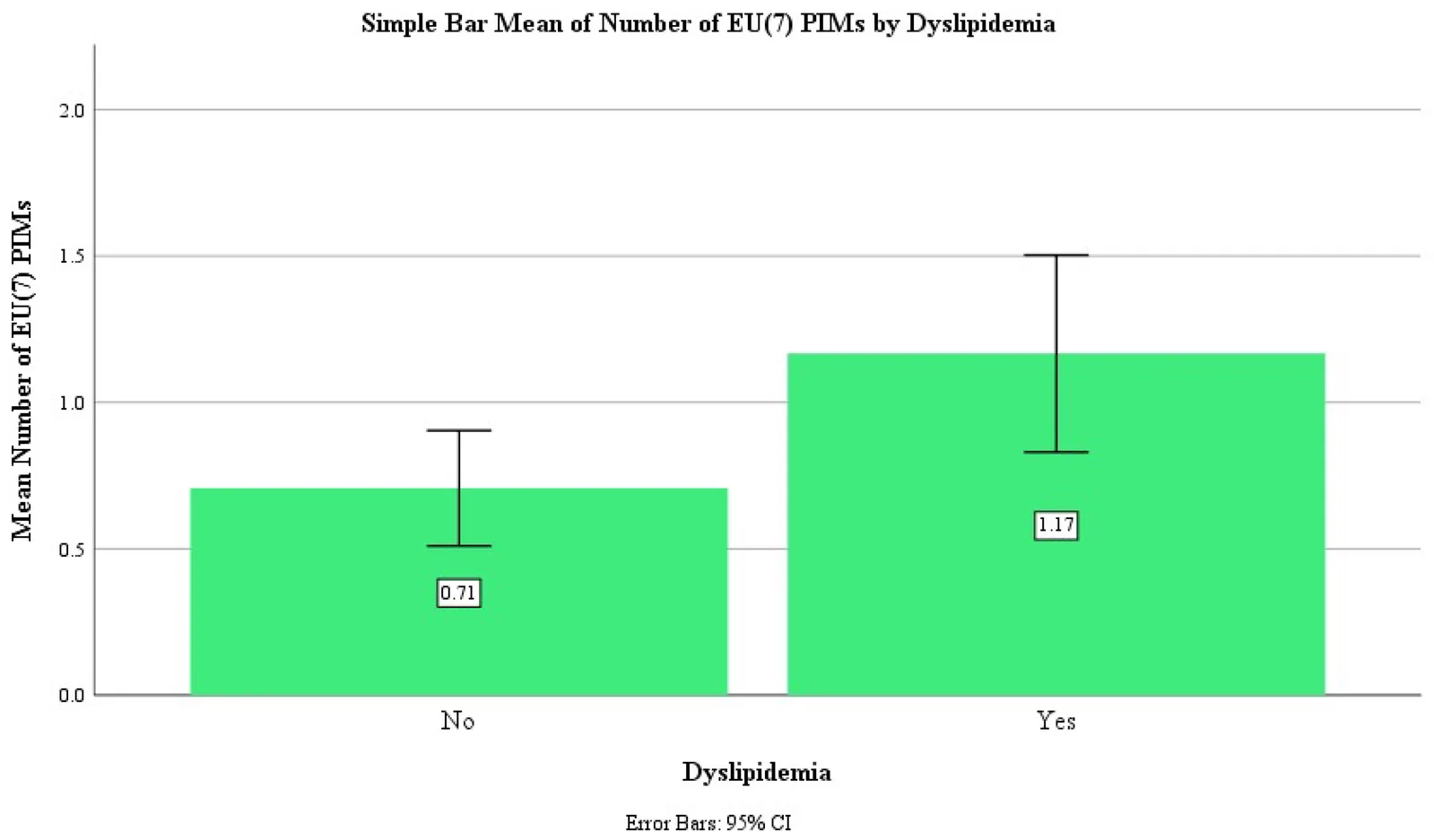
Mean number of EU(7)-PIMs among participants with and without dyslipidemia. Error bars represent 95% confidence intervals.

A one-way ANOVA revealed a statistically significant difference in EU(7)-PIM counts between participant with and without dyslipidemia, F(1, 98) = 6.32, *p* = 0.014. Individuals with dyslipidemia had higher EU(7)-PIM counts (M = 1.17, SD = 1.08) compared to those without dyslipidemia (M = 0.71, SD = 0.75) (Figure 1).

#### 3.2.4. PRISCUS Criteria

The ANOVA for PRISCUS PIMs showed no significant group differences, F(1, 98) = 0.01, *p* = 0.910. The mean number of PRISCUS PIMs was nearly identical for participants with dyslipidemia (M = 0.76, SD = 0.91) and those without (M = 0.74, SD = 0.89).

### 3.3. Associations Between Obesity and PIM Counts

#### 3.3.1. STOPP Criteria

**Figure 2:**
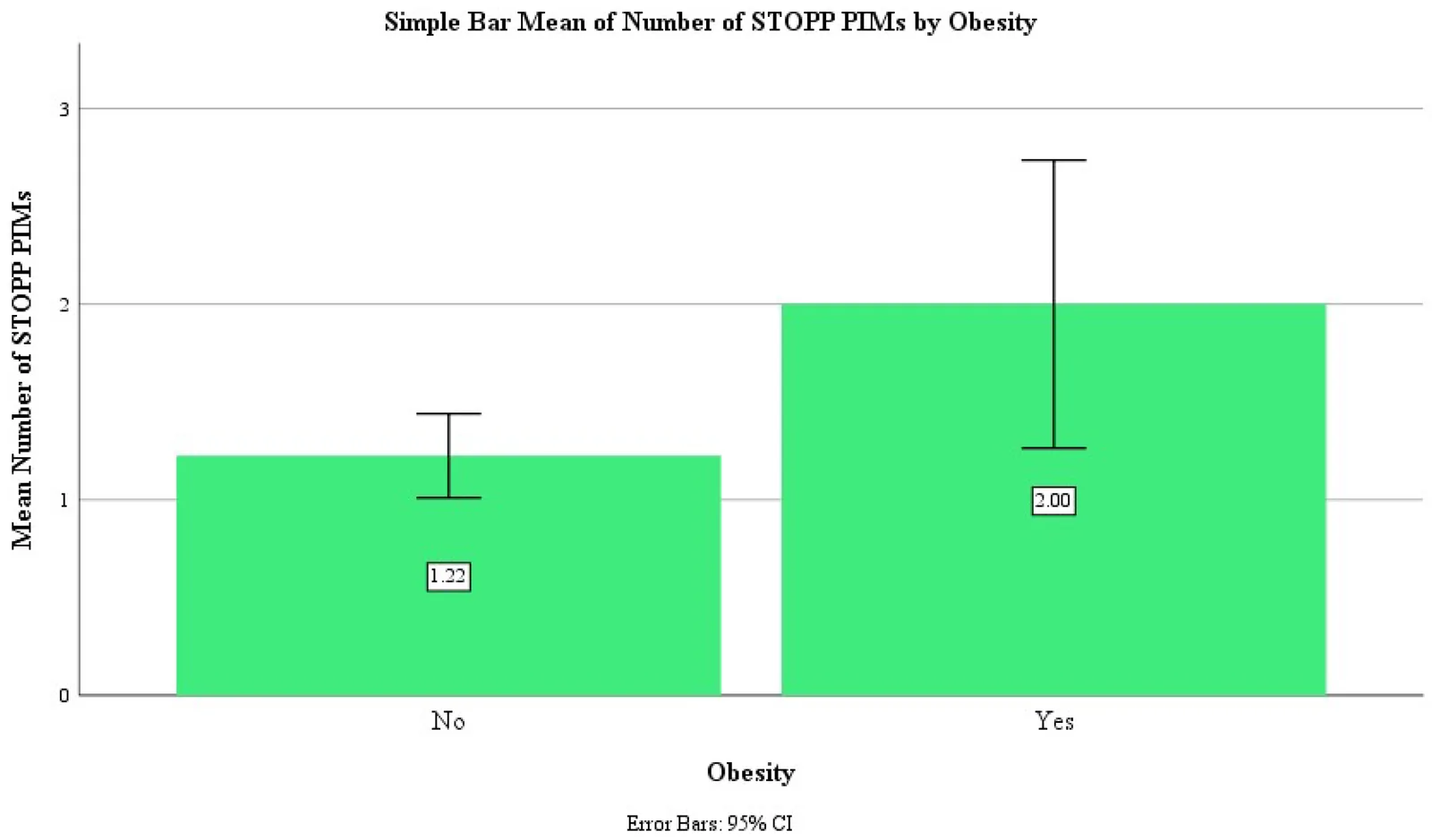
Mean number of STOPP PIMs among participants with and without obesity. Error bars represent 95% confidence intervals.

A one-way ANOVA showed a statistically significant difference in STOPP PIM counts between participants with and without obesity, F(1, 98) = 5.57, *p* = 0.020. Participants with obesity had a higher number of STOPP PIMs (M = 2.00, SD = 1.10) compared to those without obesity (M = 1.22, SD = 1.02) (Figure 2).

#### 3.3.2. Beers Criteria

**Figure 3:**
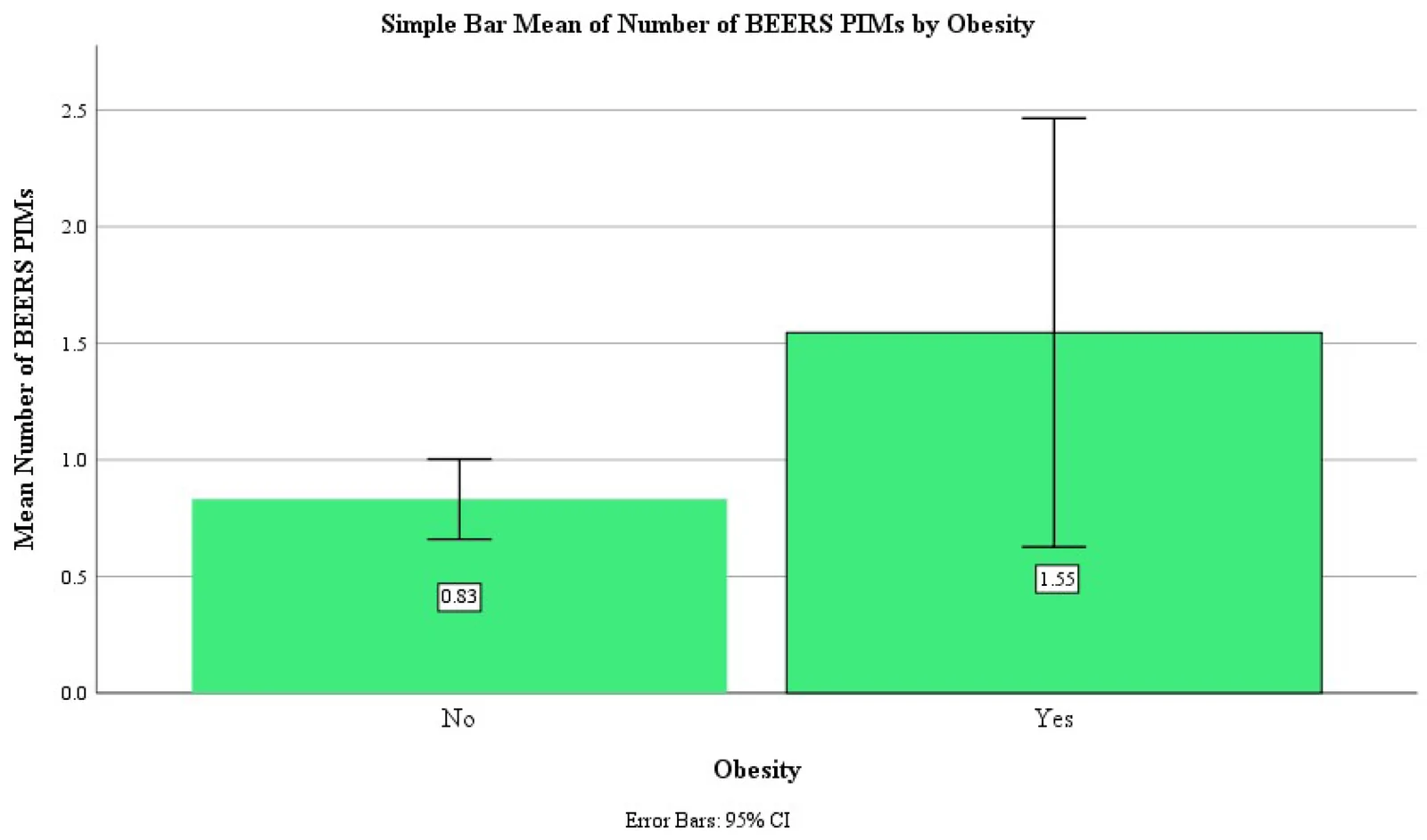
Mean number of Beers PIMs among participants with and without obesity. Error bars represent 95% confidence intervals.

The ANOVA for Beers PIMs also indicated a significant difference between groups, F(1, 98) = 6.34, *p* = 0.013. Individuals with obesity showed higher Beers PIM counts (M = 1.55, SD = 1.37) than non-obese participants (M = 0.83, SD = 0.82) (Figure 3).

#### 3.3.3. EU(7)-PIM Criteria

**Figure 4:**
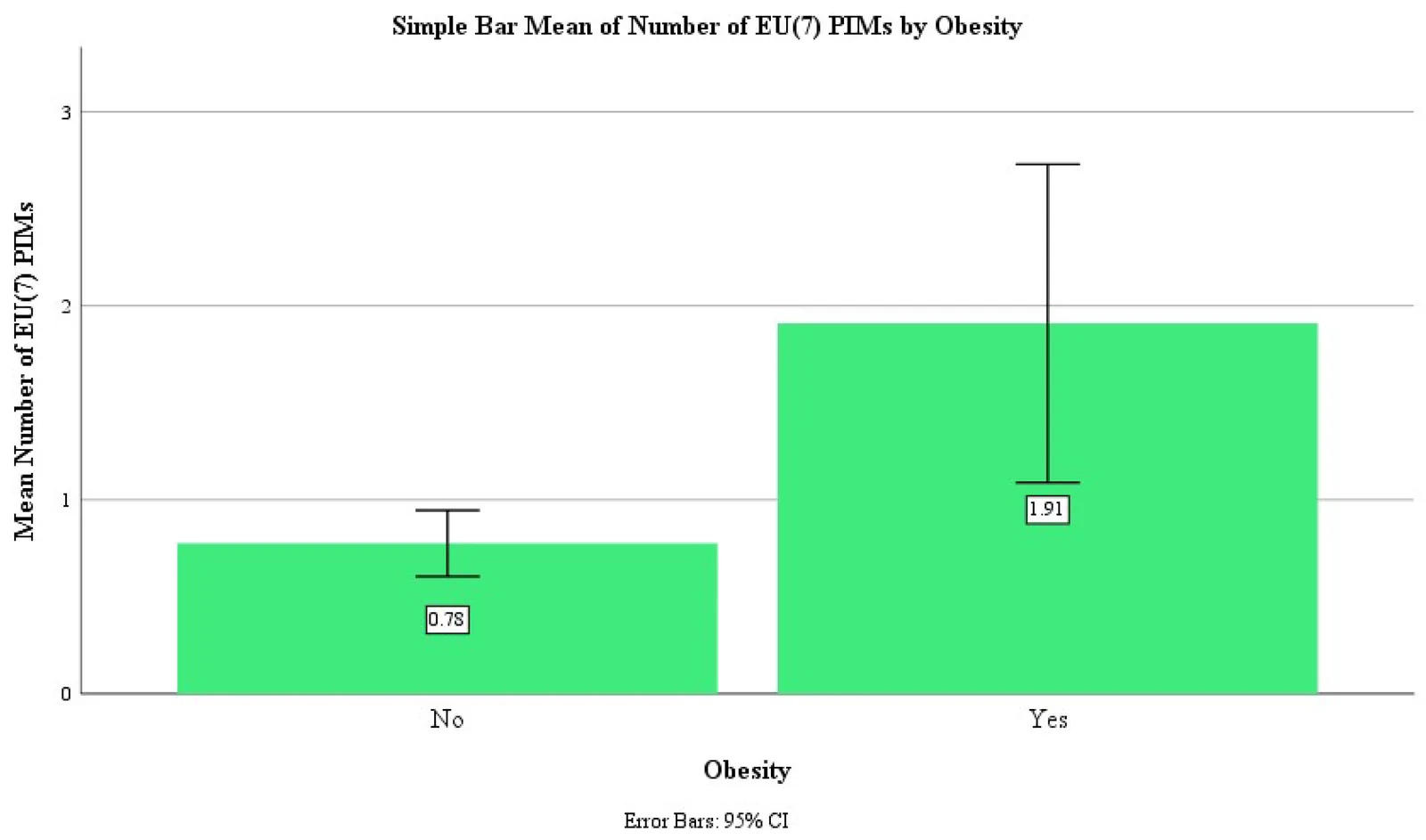
Mean number of EU(7)-PIMs among participants with and without obesity. Error bars represent 95% confidence intervals.

A one-way ANOVA revealed a significant difference in EU(7)-PIM counts between participants with and without obesity, F(1, 98) = 17.03, *p* < 0.001. Obese participants had substantially higher EU(7)-PIM counts (M = 1.91, SD = 1.22) compared to non-obese participants (M = 0.78, SD = 0.81) (Figure 4).

Given the small number of participants with obesity, a bootstrap sensitivity analysis was performed for the mean number of EU(7)-PIMs. Among participants with obesity, the mean number of EU(7)-PIMs was 1.91 (95% CI: 1.09–2.73). Based on 1000 bootstrap samples, the bias-corrected and accelerated 95% confidence interval was 1.20–2.57.

#### 3.3.4. PRISCUS Criteria

**Figure 5:**
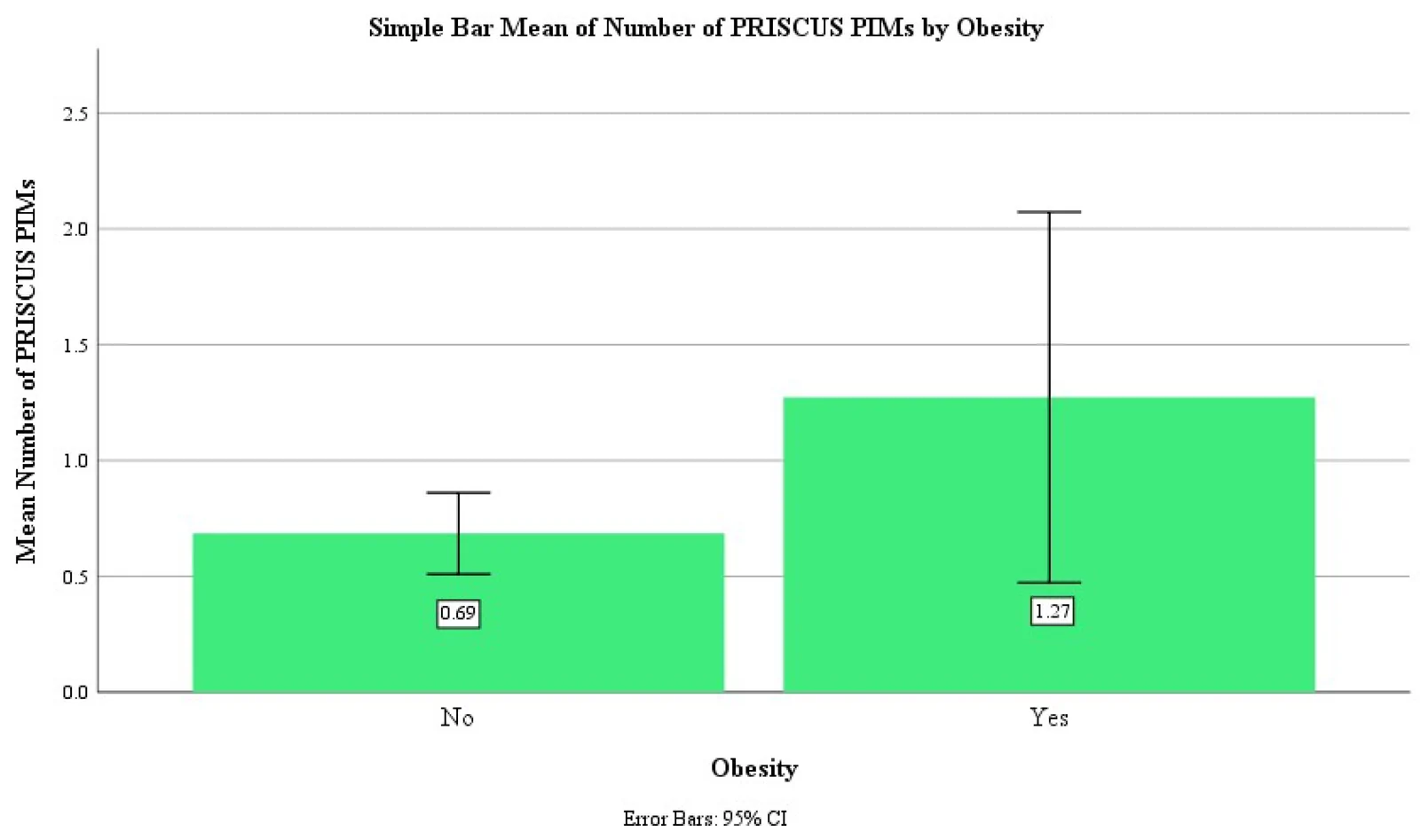
Mean number of PRISCUS PIMs among participants with and without obesity. Error bars represent 95% confidence intervals.

The ANOVA for PRISCUS PIMs also showed a significant group difference, F(1, 98) = 4.39, *p* = 0.039. Participants with obesity had a higher number of PRISCUS PIMs (M = 1.27, SD = 1.19) than those without obesity (M = 0.69, SD = 0.83) (Figure 5).

### 3.4. Associations Between Type 2 Diabetes Mellitus and PIM Counts

#### 3.4.1. STOPP Criteria

**Figure 6:**
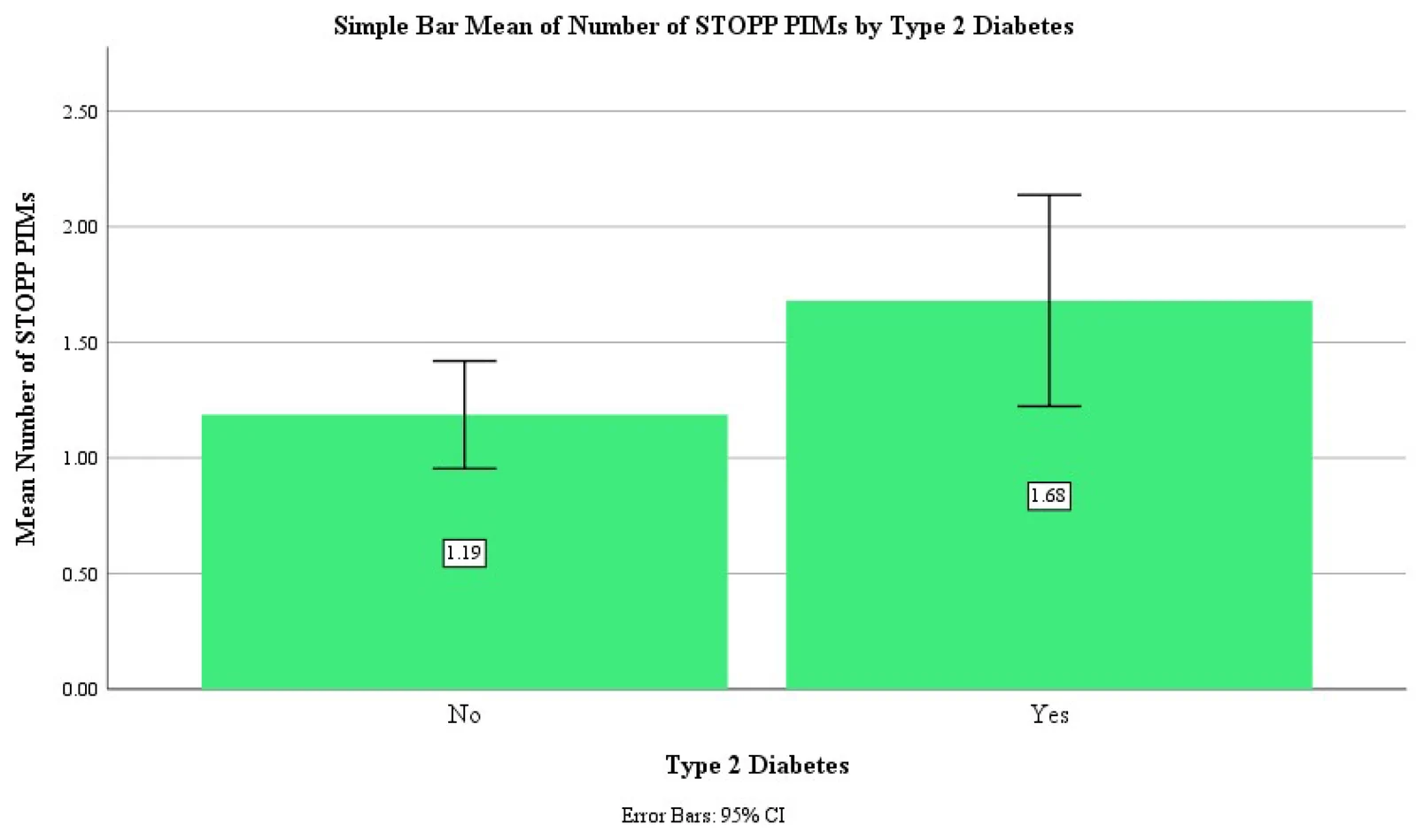
Mean number of STOPP PIMs among participants with and without Type 2 diabetes. Error bars represent 95% confidence intervals.

A one-way ANOVA showed a statistically significant difference in STOPP PIM counts between participants with and without type 2 diabetes mellitus, F(1, 98) = 4.27, *p* = 0.042. Participants with diabetes had a higher number of STOPP PIMs (M = 1.68, SD = 1.11) compared to those without diabetes (M = 1.19, SD = 1.01) (Figure 6).

#### 3.4.2. Beers Criteria

The ANOVA for Beers PIMs indicated no statistically significant difference between groups, F(1, 98) = 3.46, *p* = 0.066. Although the diabetes group showed a higher number of Beers PIMs (M = 1.20, SD = 1.08) than the non-diabetes group (M = 0.81, SD = 0.83), this difference did not reach the 0.05 threshold for significance.

#### 3.4.3. EU(7)-PIM Criteria

**Figure 7:**
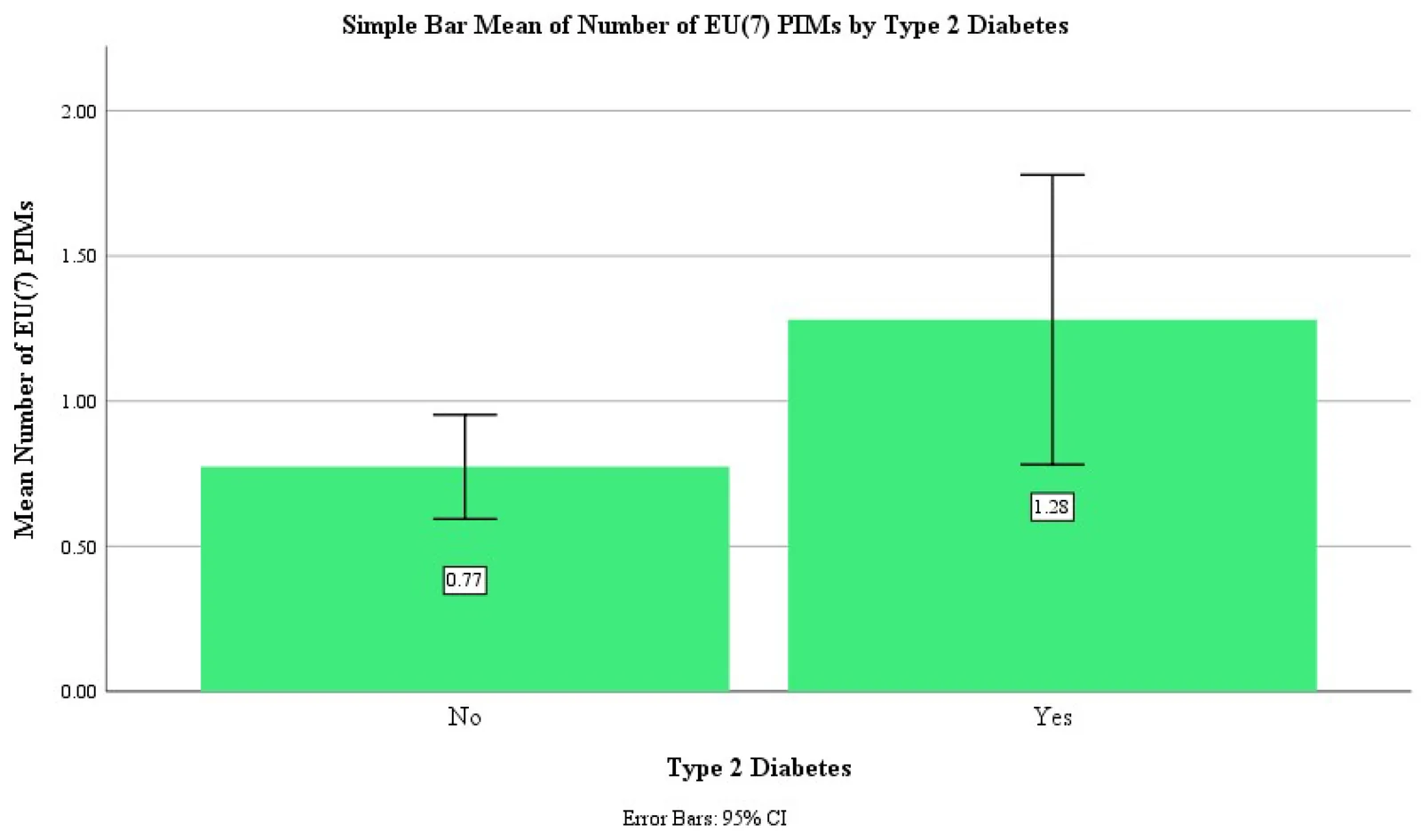
Mean number of EU(7)-PIMs among participants with and without Type 2 diabetes. Error bars represent 95% confidence intervals.

A one-way ANOVA showed a statistically significant difference in EU(7)-PIM counts between participants with and without type 2 diabetes mellitus, F(1, 98) = 5.88, *p* = 0.017. Individuals with type 2 diabetes had higher EU(7)-PIM counts (M = 1.28, SD = 1.21) compared to those without diabetes (M = 0.77, SD = 0.78) (Figure 7).

#### 3.4.4. PRISCUS Criteria

The ANOVA for PRISCUS PIMs showed no statistically significant difference between groups, F(1, 98) = 0.71, *p* = 0.403. The mean number of PRISCUS PIMs did not differ between participants with diabetes (M = 0.88, SD = 1.05) and those without diabetes (M = 0.71, SD = 0.84).

### 3.5. Associations Between Sex and PIM Counts

#### 3.5.1. STOPP Criteria

A one-way ANOVA showed no statistically significant difference in the number of STOPP PIMs between males and females, F(1, 98) = 0.79, *p* = 0.377. The mean number of STOPP PIMs did not differ significantly between males (M = 1.20, SD = 1.05) and females (M = 1.39, SD = 1.06).

#### 3.5.2. Beers Criteria

The ANOVA for Beers PIMs indicated no statistically significant difference between male and female participants, F(1, 98) = 0.80, *p* = 0.374. The mean number of Beers PIMs was 0.82 (SD = 0.87) in males and 0.98 (SD = 0.94) in females.

#### 3.5.3. EU(7)-PIM Criteria

There was no statistically significant sex difference in the number of EU(7)-PIMs, F(1, 98) = 1.49, *p* = 0.225. Males had a mean of 0.77 (SD = 0.89), while females had a mean of 1.00 (SD = 0.95).

#### 3.5.4. PRISCUS Criteria

**Figure 8:**
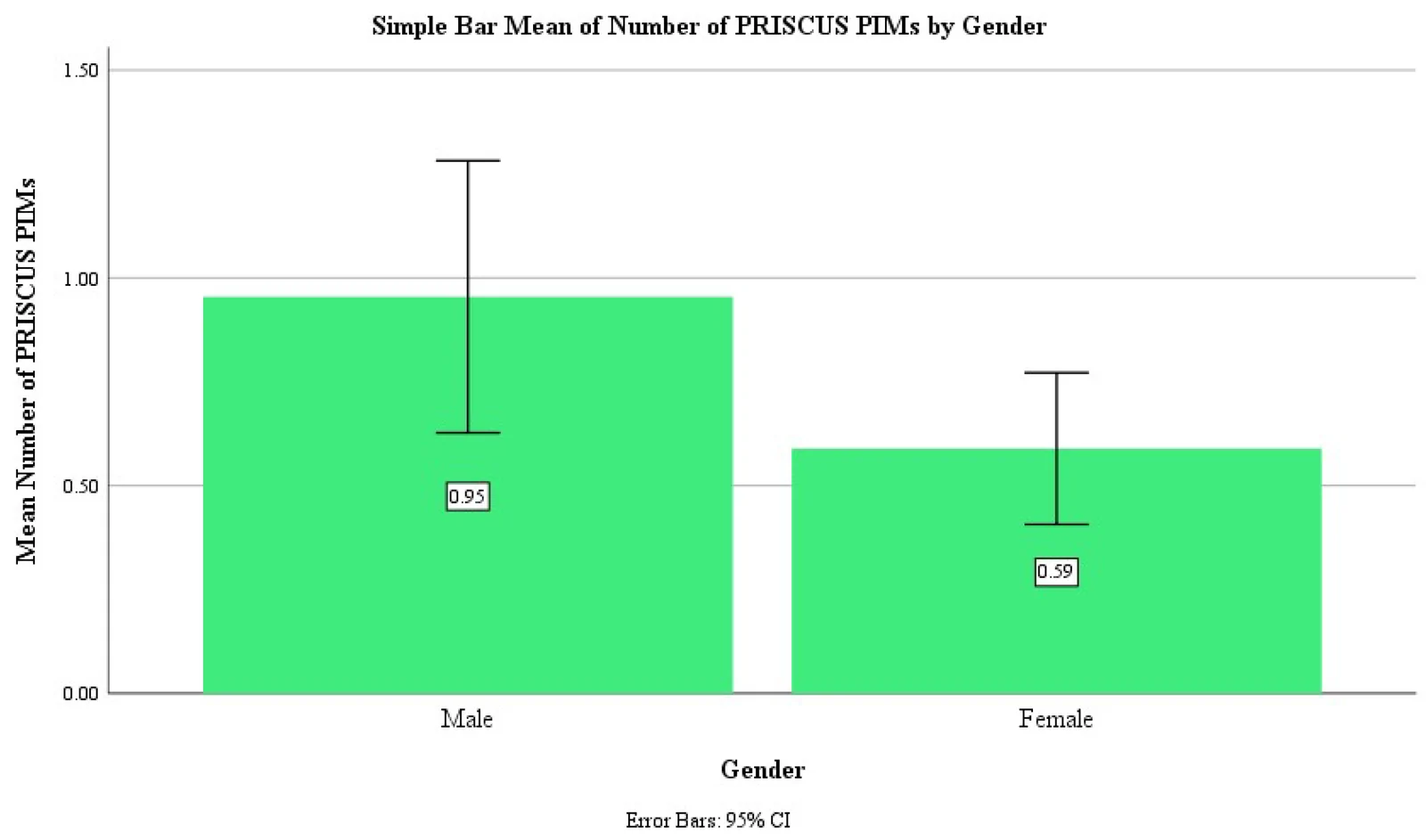
Mean number of PRISCUS PIMs among male and female participants. Error bars represent 95% confidence intervals.

A one-way ANOVA showed a statistically significant difference in PRISCUS PIM counts between male and female participants, F(1, 98) = 4.27, *p* = 0.041. Male participants had a higher mean number of PRISCUS PIMs (M = 0.95, SD = 1.08) than female participants (M = 0.59, SD = 0.68) (Figure 8).

### 3.6. Bonferroni Correction for Multiple Comparisons

To account for the risk of type I error associated with the 16 one-way ANOVA comparisons performed across the four clinical variables and four PIM criteria, a Bonferroni correction was applied. The adjusted significance threshold was α = 0.003125 (0.05/16). Following this correction, the association between obesity and EU(7)-PIM counts remained statistically significant (*p* < 0.001), representing the only association that retained statistical significance among the 16 comparisons. All other associations did not meet the Bonferroni-adjusted significance threshold.

### 3.7. Comparison of PIM Criteria

Because all four PIM criteria were applied to the same participants, a repeated-measures analysis was conducted to compare the mean number of PIMs identified by STOPP, Beers, EU(7)-PIM, and PRISCUS. Mauchly’s test indicated a violation of the assumption of sphericity (*p* = 0.001); therefore, the Greenhouse–Geisser correction was applied. The analysis showed a statistically significant difference in mean PIM counts across the four criteria, F(2.631, 260.469) = 17.247, *p* < 0.001, partial η^2^ = 0.148.

The mean number of PIMs was highest according to the STOPP criteria (M = 1.31, SD = 1.05), followed by Beers (M = 0.91, SD = 0.91), EU(7)-PIM (M = 0.90, SD = 0.93), and PRISCUS (M = 0.75, SD = 0.89). Bonferroni-adjusted pairwise comparisons showed that STOPP identified significantly more PIMs than Beers (mean difference = 0.40, *p* < 0.001), EU(7)-PIM (mean difference = 0.41, *p* < 0.001), and PRISCUS (mean difference = 0.56, *p* < 0.001). No statistically significant differences were observed between Beers and EU(7)-PIM (*p* = 1.000), Beers and PRISCUS (*p* = 0.172), or EU(7)-PIM and PRISCUS (*p* = 0.375) (Table 1).

Regarding the presence of at least one PIM, 73% of participants had at least one STOPP PIM, compared with 62% according to the Beers criteria, 60% according to the EU(7)-PIM criteria, and 53% according to the PRISCUS criteria.

To further assess the overlap between the four PIM criteria, the presence of at least one PIM was compared pairwise between criteria. The greatest overlap in PIM presence was observed between STOPP and Beers, while the lowest overlap was observed between Beers and PRISCUS. Table 2 presents the two concordant categories: participants identified as having at least one PIM according to both criteria and participants with no PIM according to either criterion. Participants identified as having at least one PIM according to only one of the two criteria were considered discordant classifications. Overall, the criteria showed substantial overlap in the identification of patients with at least one PIM, although some patients were identified by only one of the criteria.

Agreement between the four PIM criteria was assessed using Cohen’s kappa. All pairwise comparisons showed statistically significant agreement, with kappa values indicating substantial agreement between the criteria (Table 3).

### 3.8. Primary Poisson Regression Analyses

Given the count nature of the PIM outcomes, Poisson regression models were used as the primary regression analyses to examine the associations between obesity, type 2 diabetes mellitus, dyslipidemia, sex, and PIM counts according to each criterion. The goodness-of-fit statistics indicated no evidence of substantial overdispersion across the four models, with Deviance/df values ranging from 1.001 to 1.113 and Pearson Chi-Square/df values ranging from 0.821 to 0.979.

#### 3.8.1. STOPP Criteria

A Poisson regression model was conducted to examine whether obesity, type 2 diabetes, dyslipidemia, and sex were associated with the number of STOPP PIMs. The analysis showed no statistically significant associations for obesity (B = −0.34, SE = 0.26, *p* = 0.204), type 2 diabetes (B = −0.19, SE = 0.21, *p* = 0.378), dyslipidemia (B = −0.16, SE = 0.19, *p* = 0.386), or sex (B = −0.12, SE = 0.18, *p* = 0.488).

#### 3.8.2. Beers Criteria

A Poisson regression model was conducted to examine whether obesity, type 2 diabetes, dyslipidemia, and sex were associated with the number of Beers PIMs. None of the examined predictors showed a statistically significant association with Beers PIM counts. Obesity was not significantly associated with the number of Beers PIMs (B = −0.50, SE = 0.31, *p* = 0.105), while type 2 diabetes (B = −0.20, SE = 0.26, *p* = 0.423), dyslipidemia (B = −0.05, SE = 0.23, *p* = 0.812), and sex (B = −0.18, SE = 0.22, *p* = 0.418) were also not significant predictors.

#### 3.8.3. EU(7)-PIM Criteria

A Poisson regression model revealed a statistically significant association between obesity and the number of EU(7)-PIMs. Compared with participants with obesity, participants without obesity had significantly lower EU(7)-PIM counts (B = −0.72, SE = 0.29, *p* = 0.012, 95% CI [−1.29, −0.16]). Type 2 diabetes (B = −0.14, SE = 0.26, *p* = 0.578), dyslipidemia (B = −0.30, SE = 0.23, *p* = 0.187), and sex (B = −0.23, SE = 0.22, *p* = 0.300) were not significantly associated with the number of EU(7)-PIMs.

#### 3.8.4. PRISCUS Criteria

A Poisson regression model was conducted to examine associations between obesity, type 2 diabetes, dyslipidemia, sex, and the number of PRISCUS PIMs. Obesity was not significantly associated with PRISCUS PIM counts (B = −0.60, SE = 0.34, *p* = 0.079), nor were type 2 diabetes (B = −0.05, SE = 0.30, *p* = 0.878) or dyslipidemia (B = 0.04, SE = 0.25, *p* = 0.885). Sex showed a statistically significant association with PRISCUS PIM counts (B = 0.48, SE = 0.23, *p* = 0.043), with males having higher PRISCUS PIM counts than females.

### 3.9. Medication Burden and PIM Counts

Because the number of medications may influence the number of PIMs identified, additional Poisson regression models were conducted to examine the association between the number of distinct medications per patient and the number of PIMs identified by each criterion. The number of distinct medications was significantly associated with the number of STOPP PIMs (B = 0.10, SE = 0.02, *p* < 0.001), Beers PIMs (B = 0.09, SE = 0.02, *p* < 0.001), EU(7)-PIMs (B = 0.09, SE = 0.02, *p* < 0.001), and PRISCUS PIMs (B = 0.07, SE = 0.03, *p* = 0.008). For each additional distinct medication, the expected number of PIMs increased by approximately 10.5% for STOPP, 9.0% for Beers, 9.2% for EU(7), and 7.7% for PRISCUS (Table 4).

### 3.10. Adjusted Poisson Regression Analyses

Adjusted Poisson regression models were performed separately for each PIM criterion to assess the independent associations of obesity, type 2 diabetes mellitus, dyslipidemia, sex, age, multimorbidity, and medication burden with PIM counts. The number of distinct chronic diagnoses per patient was used as an indicator of multimorbidity, while the number of distinct medications per patient represented medication burden.

#### 3.10.1. STOPP PIMs

The adjusted model was statistically significant (likelihood ratio χ^2^ = 48.811, df = 7, *p* < 0.001). Medication burden was significantly associated with STOPP PIM counts (B = 0.111, SE = 0.0273, 95% CI: 0.058–0.165, *p* < 0.001). Multimorbidity was also significantly associated with STOPP PIM counts (B = 0.079, SE = 0.0400, 95% CI: 0.000–0.157, *p* = 0.049). Obesity was not significantly associated with STOPP PIM counts after adjustment (B = −0.545, SE = 0.3037, 95% CI: −1.140–0.051, *p* = 0.073). Age, type 2 diabetes, dyslipidemia, and sex were not significantly associated with STOPP PIM counts. Model fit was adequate, with Deviance/df = 0.730 and Pearson Chi-Square/df = 0.730.

#### 3.10.2. Beers PIMs

The adjusted Beers model was statistically significant (likelihood ratio χ^2^ = 24.214, df = 7, *p* = 0.001). Medication burden was significantly associated with the number of Beers PIMs (B = 0.064, SE = 0.0268, 95% CI: 0.012–0.117, *p* = 0.016). Obesity showed a borderline association with Beers PIM counts (B = −0.580, SE = 0.2977, 95% CI: −1.163–0.004, *p* = 0.052), but did not reach conventional statistical significance. Multimorbidity (B = 0.047, 95% CI: −0.030–0.124, *p* = 0.228), age (*p* = 0.524), type 2 diabetes (*p* = 0.868), dyslipidemia (*p* = 0.851), and sex (*p* = 0.326) were not significantly associated with Beers PIM counts. Model fit was adequate, with Deviance/df = 0.701 and Pearson Chi-Square/df = 0.701.

#### 3.10.3. EU(7)-PIMs

The adjusted EU(7)-PIM model was statistically significant (likelihood ratio χ^2^ = 40.102, df = 7, *p* < 0.001). Obesity remained significantly associated with EU(7)-PIM counts (B = −0.893, SE = 0.2797, 95% CI: −1.441 to −0.344, *p* = 0.001). Medication burden was also significantly associated with EU(7)-PIM counts (B = 0.060, SE = 0.0252, 95% CI: 0.010–0.109, *p* = 0.018). Multimorbidity showed no statistically significant association (B = 0.064, 95% CI: −0.008–0.136, *p* = 0.084). Age, type 2 diabetes, dyslipidemia, and sex were not significantly associated with EU(7)-PIM counts. Model fit was adequate, with Deviance/df = 0.619 and Pearson Chi-Square/df = 0.619.

#### 3.10.4. PRISCUS PIMs

The adjusted PRISCUS model was statistically significant (likelihood ratio χ^2^ = 20.536, df = 7, *p* = 0.005). Sex was significantly associated with PRISCUS PIM counts (B = 0.360, SE = 0.1658, 95% CI: 0.035–0.685, *p* = 0.030). Medication burden was not significantly associated with PRISCUS PIM counts after adjustment (B = 0.038, SE = 0.0267, 95% CI: −0.015–0.090, *p* = 0.157). Similarly, obesity (B = −0.471, SE = 0.2968, 95% CI: −1.053–0.110, *p* = 0.112) and multimorbidity (B = 0.069, SE = 0.0390, 95% CI: −0.007–0.146, *p* = 0.077) were not significantly associated with PRISCUS PIM counts. Age, type 2 diabetes, and dyslipidemia were also not significantly associated with PRISCUS PIM counts. Model fit was adequate, with Deviance/df = 0.697 and Pearson Chi-Square/df = 0.697.

Overall, medication burden was independently associated with higher PIM counts according to STOPP, Beers, and EU(7)-PIM criteria, whereas multimorbidity was associated with STOPP PIM counts only. Obesity remained independently associated with EU(7)-PIM counts but not with STOPP, Beers, or PRISCUS PIM counts after adjustment. Sex was significantly associated with PRISCUS PIM counts, while age was not significantly associated with PIM counts according to any of the four criteria (Table 5).

### 3.11. Supplementary Linear Regression Analyses

#### 3.11.1. Association Between Age and PIM Counts

As a supplementary sensitivity analysis, simple linear regression was used to examine the association between age and PIM counts according to each of the four criteria. Age was not significantly associated with STOPP PIM counts (β = 0.078, *p* = 0.442), Beers PIM counts (β = 0.045, *p* = 0.654), EU(7)-PIM counts (β = 0.097, *p* = 0.336), or PRISCUS PIM counts (β = 0.032, *p* = 0.755). In addition, supplementary multivariable linear regression analyses were conducted to examine the associations of obesity, type 2 diabetes mellitus, dyslipidemia, and sex with PIM counts according to the four explicit criteria.

#### 3.11.2. STOPP

The first regression model predicting STOPP PIM counts was not statistically significant, F(4, 95) = 2.23, *p* = 0.072, indicating that obesity, diabetes, dyslipidemia, and sex collectively did not predict the number of STOPP PIMs. Because the overall model was nonsignificant, individual predictors were not interpreted.

#### 3.11.3. Beers

The model predicting Beers PIM counts was also nonsignificant, F(4, 95) = 2.02, *p* = 0.097, suggesting that the predictors did not explain a significant proportion of the variance in the number of Beers PIMs. Individual predictors were not interpreted.

#### 3.11.4. PRISCUS

Similarly, the regression model for PRISCUS PIM counts was not statistically significant, F(4, 95) = 2.19, *p* = 0.076. Given the nonsignificant overall model, individual predictors were not interpreted.

#### 3.11.5. EU(7)-PIM

**Figure 9:**
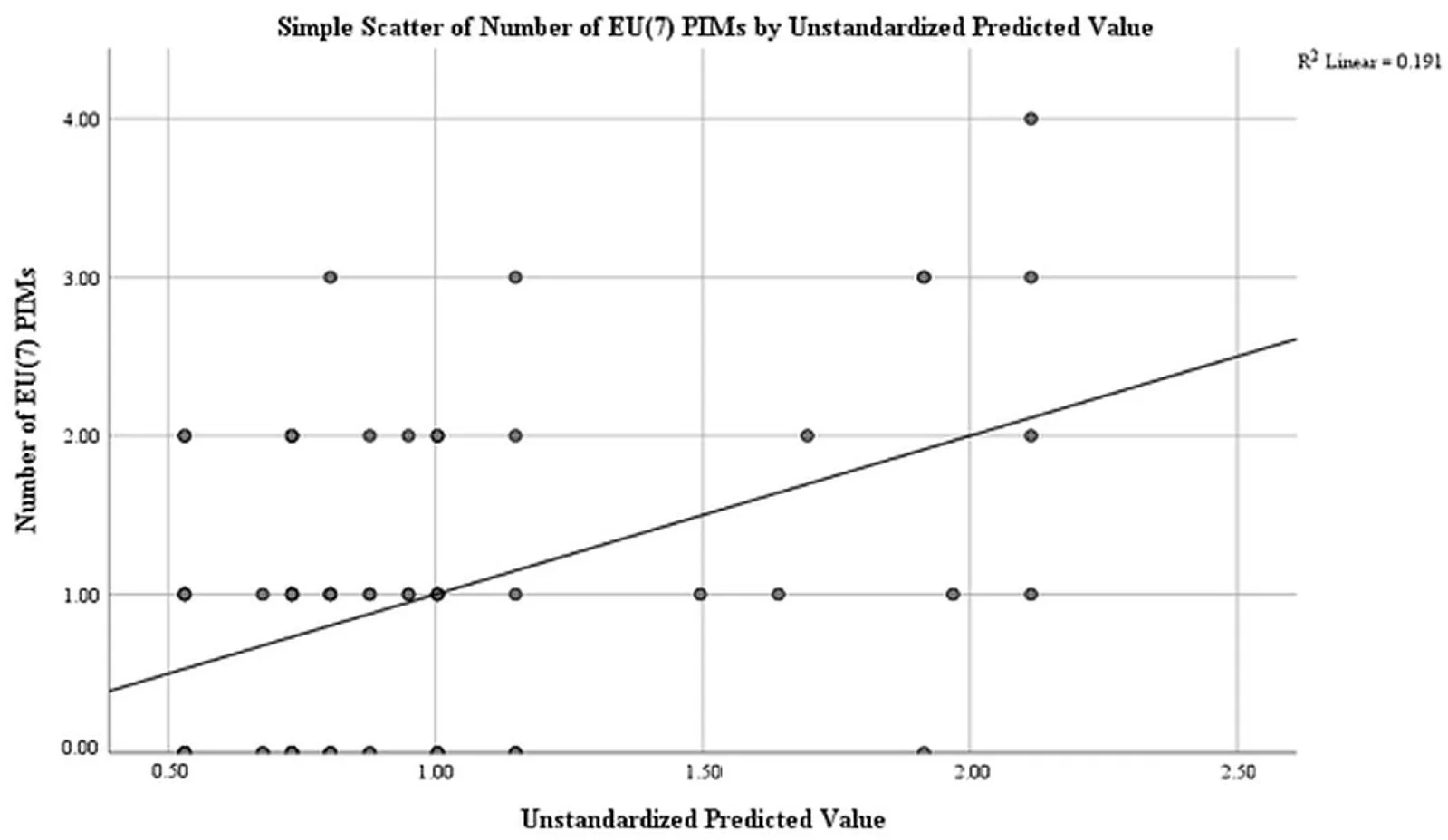
Observed and model-predicted number of EU(7)-PIMs from the multiple regression model. The *X*-axis represents the unstandardized predicted values (PRE), which reflect the estimated number of EU(7)-PIMs generated by the regression equation after accounting for all model predictors. The fitted line illustrates the relationship between the observed and predicted values.

In contrast, the model predicting EU(7)-PIM counts was statistically significant, F(4, 95) = 5.62, *p* < 0.001, explaining 19.1% of the variance in EU(7)-PIM counts (R = 0.44, R^2^ = 0.191, adjusted R^2^ = 0.157) (Figure 9).

To further examine the association between obesity and EU(7)-PIM counts, a simple linear regression was also conducted with obesity as the sole independent variable. This model was statistically significant, F(1, 98) = 17.03, *p* < 0.001, and obesity alone accounted for 14.8% of the variance in the number of EU(7)-PIMs (R = 0.385, R^2^ = 0.148, adjusted R^2^ = 0.139). Obesity was significantly associated with a higher number of EU(7)-PIMs (B = 1.13, SE = 0.28, β = 0.39, t = 4.13, *p* < 0.001, 95% CI [0.59, 1.68]).

## 4. Discussion

The present study examined PIM identification according to four explicit screening criteria and explored associations between PIM counts and selected patient characteristics in older adults attending a single primary care practice. Several associations were observed in the initial analyses; however, after correction for multiple comparisons, only the association between obesity and EU(7)-PIM counts remained statistically significant. In the adjusted Poisson models, medication burden showed the most consistent association with PIM counts, with significant associations observed for STOPP, Beers, and EU(7)-PIM. Multimorbidity was associated with STOPP PIM counts, obesity with EU(7)-PIM counts, and sex with PRISCUS PIM counts, whereas age, type 2 diabetes mellitus, and dyslipidemia were not independently associated with PIM counts. These results should be considered in the context of the exploratory nature of the study, its single-practice setting, and the relatively small sample size.

Medication burden emerged as the factor most consistently associated with PIM counts in the adjusted analyses. This finding is consistent with previous evidence showing that polypharmacy is closely associated with potentially inappropriate prescribing in older adults [6,7,28]. As the number of medications increases, therapeutic regimens become more complex, which may in turn increase the likelihood of exposure to medicines considered potentially inappropriate by explicit screening criteria. In the present study, medication burden remained associated with PIM counts according to three of the four screening tools, highlighting the relevance of overall pharmacotherapy complexity when evaluating medication appropriateness in older adults.

Obesity was associated with higher PIM counts according to all four criteria in the initial one-way ANOVA analyses. After correction for multiple comparisons, however, only the association with EU(7)-PIM remained statistically significant, and this association was also observed in the adjusted Poisson model. This finding is clinically plausible, as obesity has been associated with greater multimorbidity and medication burden in older populations [28,29,30]. Nevertheless, obesity was documented in only 11 participants in the present study, and estimates involving this subgroup should therefore be interpreted cautiously. Although the bootstrap sensitivity analysis yielded a similar estimate for the mean EU(7)-PIM count, the small size of the obesity subgroup limits the precision and generalizability of this finding.

Type 2 diabetes mellitus was associated with higher STOPP and EU(7)-PIM counts in the initial analyses, although these associations did not remain statistically significant after correction for multiple comparisons. Furthermore, type 2 diabetes mellitus was not independently associated with PIM counts in the adjusted Poisson models. Previous studies have reported substantial PIM exposure among older adults with diabetes and have shown that PIM use may vary according to clinical and demographic characteristics [31,32,33]. Older adults with type 2 diabetes frequently present with a considerable burden of comorbidity and complex pharmacotherapy, factors that may contribute to potentially inappropriate medication use [28,34]. Therefore, the present findings should not be interpreted as evidence that diabetes is unrelated to PIM counts but rather as indicating that an independent association was not demonstrated in this exploratory sample.

Dyslipidemia was associated with higher EU(7)-PIM counts in the initial analysis, but this association did not remain statistically significant after correction for multiple comparisons and was not observed in the adjusted Poisson model. Previous comparisons of explicit PIM criteria have shown that the medications and clinical situations identified may differ between screening tools [35,36]. The association observed with EU(7)-PIM in the initial analysis should therefore be regarded as an exploratory finding rather than evidence of an independent relationship between dyslipidemia and PIM counts.

Sex showed a criterion-specific association with PIM counts. No statistically significant differences between male and female participants were observed for STOPP, Beers, or EU(7)-PIM, whereas males had higher PRISCUS PIM counts in the initial analysis. This association also remained statistically significant in the adjusted PRISCUS Poisson model. Previous studies have frequently reported a higher prevalence of PIM use among older women, including analyses based on PRISCUS and other explicit screening criteria [32,37]. The different pattern observed in the present study may reflect differences in the medicines captured by individual criteria [38], as well as local prescribing patterns or characteristics of the relatively small sample. This finding should therefore be interpreted cautiously.

The within-participant comparison of the four screening criteria also demonstrated differences in PIM identification. STOPP identified the highest mean number of PIMs and the highest proportion of participants with at least one PIM, whereas PRISCUS identified the lowest. Pairwise comparisons showed significantly higher PIM counts with STOPP than with Beers, EU(7)-PIM, and PRISCUS, while no statistically significant differences were observed among the latter three criteria. At the same time, the criteria showed considerable overlap in the participants identified as having at least one PIM, with pairwise kappa coefficients ranging from 0.506 to 0.777. Previous studies have likewise reported differences and incomplete concordance between explicit PIM criteria [35,36,38], which may reflect differences in their scope and in the medicines or clinical situations covered by each tool. Because no external reference standard was used in the present study, these findings cannot establish that one criterion is more sensitive or superior to another. Rather, they indicate that PIM identification may vary according to the criterion applied.

From a clinical perspective, the results support the relevance of medication burden when reviewing pharmacotherapy in older adults. The differences observed between explicit criteria also indicate that individual screening tools may capture partly different aspects of potentially inappropriate medication use. However, given the exploratory nature of the present study, these findings are insufficient to support preferential use of one criterion or the routine combined application of several tools. Larger multicenter studies involving more diverse primary care populations are needed to determine whether the associations and between-criterion differences observed here are reproducible.

A strength of this study is the simultaneous application of four explicit PIM criteria, including the updated STOPP v3 and PRISCUS v2 tools, in a primary care population of older adults. This approach allowed direct within-participant comparisons of PIM counts, the proportion of participants identified with at least one PIM, and agreement between the different criteria.

Several limitations should be considered when interpreting these findings. First, the study was conducted in a single family medicine practice and used a non-probability sampling approach, which, together with the relatively small sample size of 100 participants, may limit the generalizability of the results. No formal a priori sample-size calculation was performed, and the analyses should therefore be regarded as exploratory. In particular, obesity was documented in only 11 participants, resulting in limited precision for estimates involving this subgroup despite the additional bootstrap sensitivity analysis. The overall sample size also constrained the number of variables that could reasonably be included in the multivariable analyses, and associations close to the conventional threshold for statistical significance should be interpreted cautiously. Patients receiving only one medication were excluded because the database was designed to investigate medication-related issues among older adults receiving more than one pharmacological treatment; consequently, the findings may not be directly applicable to older adults with a lower medication burden. Obesity, type 2 diabetes mellitus, and dyslipidemia were defined according to diagnoses documented in the available medical records rather than through study-specific clinical or laboratory reassessment, which may have resulted in some degree of misclassification. PIM identification was performed by a single clinical pharmacist and was not independently reviewed by a second assessor, which represents an additional limitation of the study. In addition, the retrospective cross-sectional design precludes conclusions regarding temporal or causal relationships. Finally, medication exposure was determined from treatments documented in the available medical records, and some medicines prescribed outside the primary care setting may not have been fully captured.

## 5. Conclusions

The number of potentially inappropriate medications identified varied with the explicit criteria applied: the STOPP criteria yielded the highest mean number of PIMs, and the PRISCUS criteria the lowest. In the adjusted analyses, medication burden was the factor most consistently associated with PIM counts, with significant associations observed for STOPP, Beers, and EU(7)-PIM. Obesity remained associated with EU(7)-PIM counts, multimorbidity with STOPP PIM counts, and sex with PRISCUS PIM counts, while age, type 2 diabetes mellitus, and dyslipidemia were not independently associated with PIM counts. These findings illustrate that both patient-related factors and the choice of screening criterion may influence PIM identification. Given the relatively small sample, single-practice setting, and non-probability sampling approach, the results should be considered exploratory and require confirmation in larger, multicenter primary care studies.

## Figures and Tables

**Table 1:** Pairwise comparisons of PIM counts between the four explicit criteria.

Pairwise Comparison	Mean Difference	95% CI	*p*-Value
STOPP vs. Beers	0.40	0.16–0.64	<0.001
STOPP vs. EU(7)-PIM	0.41	0.19–0.63	<0.001
STOPP vs. PRISCUS	0.56	0.30–0.82	<0.001
Beers vs. EU(7)-PIM	0.01	−0.17–0.19	1.000
Beers vs. PRISCUS	0.16	−0.03–0.35	0.172
EU(7)-PIM vs. PRISCUS	0.15	−0.06–0.36	0.375

**Table 2:** Pairwise overlap in PIM presence between the four explicit criteria.

Comparison	PIM According to Both Criteria (*n*)	No PIM According to Either Criterion (*n*)
STOPP vs. Beers	59	24
STOPP vs. EU(7)-PIM	57	24
STOPP vs. PRISCUS	51	25
Beers vs. EU(7)-PIM	54	32
Beers vs. PRISCUS	50	35
EU(7)-PIM vs. PRISCUS	51	38

**Table 3:** Pairwise agreement between the four PIM criteria based on Cohen’s kappa.

Comparison	Cohen’s Kappa	*p*-Value
STOPP vs. Beers	0.618	<0.001
STOPP vs. EU(7)-PIM	0.581	<0.001
STOPP vs. PRISCUS	0.506	<0.001
Beers vs. EU(7)-PIM	0.706	<0.001
Beers vs. PRISCUS	0.696	<0.001
EU(7)-PIM vs. PRISCUS	0.777	<0.001

**Table 4:** Poisson regression analysis of the association between the number of distinct medications and PIM counts.

PIM Criterion	B	SE	95% CI	*p*-Value
STOPP	0.100	0.020	0.061–0.140	<0.001
Beers	0.086	0.025	0.038–0.135	<0.001
EU(7)-PIM	0.088	0.025	0.039–0.137	<0.001
PRISCUS	0.074	0.028	0.019–0.128	0.008

**Table 5:** Adjusted Poisson regression analysis of factors associated with PIM counts according to STOPP, Beers, EU(7)-PIM, and PRISCUS criteria.

PIM Criterion	Predictor	B	SE	95% CI	*p*-Value
STOPP	Obesity	−0.545	0.3037	−1.140 to 0.051	0.073
STOPP	Type 2 Diabetes	0.132	0.2219	−0.303 to 0.567	0.551
STOPP	Dyslipidemia	−0.177	0.1761	−0.522 to 0.168	0.314
STOPP	Sex	−0.173	0.1697	−0.505 to 0.160	0.309
STOPP	Age	0.016	0.0156	−0.015 to 0.047	0.307
STOPP	Multimorbidity	0.079	0.0400	0.000 to 0.157	0.049
STOPP	Medication burden	0.111	0.0273	0.058 to 0.165	<0.001
Beers	Obesity	−0.580	0.2977	−1.163 to 0.004	0.052
Beers	Type 2 Diabetes	0.036	0.2175	−0.390 to 0.463	0.868
Beers	Dyslipidemia	−0.032	0.1726	−0.371 to 0.306	0.851
Beers	Sex	−0.163	0.1663	−0.489 to 0.163	0.326
Beers	Age	0.010	0.0153	−0.020 to 0.040	0.524
Beers	Multimorbidity	0.047	0.0392	−0.030 to 0.124	0.228
Beers	Medication burden	0.064	0.0268	0.012 to 0.117	0.016
EU(7)-PIM	Obesity	−0.893	0.2797	−1.441 to −0.344	0.001
EU(7)-PIM	Type 2 Diabetes	0.114	0.2043	−0.286 to 0.515	0.576
EU(7)-PIM	Dyslipidemia	−0.218	0.1621	−0.536 to 0.099	0.178
EU(7)-PIM	Sex	−0.178	0.1562	−0.485 to 0.128	0.254
EU(7)-PIM	Age	−0.010	0.0144	−0.038 to 0.018	0.496
EU(7)-PIM	Multimorbidity	0.064	0.0368	−0.008 to 0.136	0.084
EU(7)-PIM	Medication burden	0.060	0.0252	0.010 to 0.109	0.018
PRISCUS	Obesity	−0.471	0.2968	−1.053 to 0.110	0.112
PRISCUS	Type 2 Diabetes	0.189	0.2169	−0.236 to 0.614	0.384
PRISCUS	Dyslipidemia	0.071	0.1721	−0.267 to 0.408	0.682
PRISCUS	Sex	0.360	0.1658	0.035 to 0.685	0.030
PRISCUS	Age	−0.002	0.0153	−0.032 to 0.027	0.871
PRISCUS	Multimorbidity	0.069	0.0390	−0.007 to 0.146	0.077
PRISCUS	Medication burden	0.038	0.0267	−0.015 to 0.090	0.157

## Data Availability

The raw data supporting the conclusions of this article will be made available by the authors on request.

## Abbreviations

The following abbreviations are used in this manuscript:

ANOVA	Analysis of variance
INN	International nonproprietary name
M	Mean
PIM	Potentially inappropriate medication
PIP	Potentially inappropriate prescribing
PPO	Potential prescribing omission
SD	Standard deviation
START	Screening Tool to Alert to Right Treatment
STOPP	Screening Tool of Older Persons’ Prescriptions
